# Phytoplankton taxonomic and functional diversity patterns across a coastal tidal front

**DOI:** 10.1038/s41598-021-82071-0

**Published:** 2021-01-29

**Authors:** Pierre Ramond, Raffaele Siano, Sophie Schmitt, Colomban de Vargas, Louis Marié, Laurent Memery, Marc Sourisseau

**Affiliations:** 1 Sorbonne Université, CNRS-UMR7144-Station Biologique de Roscoff, Place Georges Teissier, 29688 Roscoff, France; 2 Ifremer-Centre de Brest, DYNECO/Pelagos, Technopôle Brest Iroise, 29280 Plouzané, France; 3 Department of Marine Microbiology and Biogeochemistry, NIOZ-Royal Netherlands Institute for Sea Research and Utrecht University, Den Burg, The Netherlands; 4 Research Federation for the Study of Global Ocean Systems Ecology and Evolution, FR2022/GOSEE, 3 rue Michel-Ange, 75016 Paris, France; 5 Laboratoire d’Océanographie Physique et Spatiale (LOPS), UMR 6523 Univ. Brest, CNRS, IFREMER, IRD, Plouzané, France; 6 Laboratoire des Sciences de l’Environnement MARin (LEMAR), UMR 6539 Univ. Brest, CNRS, IFREMER, IRD, Plouzané, France

**Keywords:** Community ecology, Microbial ecology, Molecular ecology, Ocean sciences

## Abstract

Oceanic physics at fine scale; e.g. eddies, fronts, filaments; are notoriously difficult to sample. However, an increasing number of theoretical approaches hypothesize that these processes affect phytoplankton diversity which have cascading effects on regional ecosystems. In 2015, we targeted the Iroise Sea (France) and evidenced the setting up of the Ushant tidal front from the beginning of spring to late summer. Seawater samples were taken during three sampling cruises and DNA-barcoding allowed us to investigate patterns of eukaryotic phytoplankton diversity across this front. First focusing on patterns of taxonomic richness, we evidenced that the front harbored a hotspot of eukaryotic phytoplankton diversity sustained throughout summer. We then detail the ecological processes leading to the formation of this hotspot by studying shifts in community composition across the Iroise Sea. Physical mixing mingled the communities surrounding the front, allowing the formation of a local ecotone, but it was cycles of disturbances and nutrient inputs over the front that allowed a decrease in competitive exclusion, which maintained a higher diversity of rare phytoplankton taxa. These processes did not select a specific ecological strategy as inferred by a trait approach coupled to our taxonomic approach. Instead the front favored higher richness within widespread strategies, resulting in functional redundancy. We detail how fine-scale ocean physics affect phytoplankton diversity and suppose that this interplay is a major control on regional ecosystems.

## Introduction

Photosynthetic microbes (i.e. phytoplankton) play key roles in marine ecosystems, driving biogeochemical cycles through their uptake of nutrients and carbon sequestration^1,2^, or producing the primary organic matter that supports marine food-webs^3,4^. The diversity of phytoplankton is of prime importance in these processes as richer phytoplankton communities are more efficient in nutrient uptake^5^, while larger phytoplankton assemblages promote the productivity and diversity of higher trophic levels^6^. Studying phytoplankton diversity is thus crucial to understand the interplay between microbes and the functioning of marine ecosystems. If previous studies have focused on phytoplankton diversity patterns at larger scale^7–9^, observations at the meso- and submeso-scale, of 100–0.1 km spatial and days to months temporal ranges^10^, remain scarce.

These studies showed that oceanic phytoplankton can be strongly affected by mesoscale (i.e. large eddies, fronts)^11–13^ and sub-mesoscale physical processes (i.e. smaller eddies, fronts, filaments)^14^. These processes have in common that they modulate the distribution of plankton, its access to resource and the resultant competition between taxa^15,16^. Despite numerous theoretical studies^17–20^, only few attempts were made to estimate the in-situ phytoplankton taxonomic diversity associated to meso and sub-mesoscale processes^13,21^, mainly because of the difficulty in locating and sampling these highly dynamic hydrographic features. Tidal fronts in coastal areas, ranging in between the sub-mesoscale and mesoscale, are recurrent^22^ and thus easier to target. They provide a great opportunity to investigate the in-situ interplay between small-scale ocean physics and the ecological processes driving phytoplankton diversity patterns.

Tidal fronts form seasonally in coastal ecosystems. In summer, the increase in atmospheric temperature warms surface seawaters, causing a stratification that prevents vertical mixing between the surface and bottom waters. In deep areas, tidal currents causes friction and mixing of the bottom water by interacting with the bathymetry but are not able to break the surface stratification^23^. Reversely, in shallow waters, the vertical mixing caused by bottom friction can reach the surface and prevents water stratification. Tidal fronts occurs at the frontier between (1) the deep, stratified, offshore water mass, and (2) the shallow, mixed, coastal water mass^24^. The sharp gradient in sea surface temperature at coastal tidal fronts makes them easier to survey^25,26^. Their development and seasonal dynamics have also been relatively well studied. As the summer progresses, both the coastal and offshore water masses become depleted in nutrients due to the uptake of phytoplankton and the reduction of nutrient inputs^27–29^. However, at fronts, tidal mixing still erodes and breaks the stratification. This results in a local upward vertical flux of nutrients from the offshore bottom water mass to the sunlit surface layer^30–32^, causing hydrographic discontinuities and outbursts of primary production over the front^23,33,34^. The effect of vertical mixing over coastal fronts is also strongly regulated by the spring/neap tide cycle (periodicity of ~ 14 days)^35^. Indeed, nutrients are mainly brought to the surface during the spring tides when tidal mixing is stronger, but it is during neap tides that re-stratification improves light availability which allows phytoplankton to grow in the vicinity of fronts^34,36^.

Phytoplankton’s community composition and diversity might undergo several ecological processes shaped by coastal tidal fronts^37^. Modulations in seawater conditions might favor different organisms with distinct environmental preferences (or niche), through the process called “Selection”. Selection might then affect the coexistence of phytoplankton species by modulating the timescale of competitive exclusion, i.e. the time needed by one selected species to outcompete the others under steady conditions^38,39^. Despite the small geographic scale at which coastal tidal fronts appear, they might also affect the movement (active or passive) and colonization of phytoplankton taxa across the offshore, frontal and coastal areas^20^, this process is called “Dispersal” (limitation or facilitation). Other ecological processes^37^, notably “Ecological Drift”; i.e. the random changes in community composition due to the inherent stochastic processes of birth, death, and reproduction; or “Diversification”; i.e. evolutionary generation of new species; are likely to have a smaller impact over coastal tidal fronts. In this study, we propose to investigate the drivers of phytoplankton diversity over a coastal tidal front.

The Ushant tidal front forms in the Iroise Sea and separates offshore waters coming from the Celtic Shelf^40^ from coastal waters moving northward along the French coast^41^. We sampled five stations distributed across this front along three time periods representing the cycle of formation and termination of the Ushant tidal front. We studied eukaryotic phytoplankton using DNA-barcoding in order to investigate diversity at a high resolution. We coupled this taxonomic approach to a trait approach to also study the functional diversity of phytoplankton. Traits represent any metric of an organism that informs its ecological strategy; i.e. strategy of resource acquisition, growth, reproduction or survival. Traits can underline the ecological strategy favored by selection processes^42,43^. We first detail patterns of taxonomy and richness across the coastal tidal front. We then make use of an approach that compares observed to random community turnovers (i.e. shifts in community composition) to infer the dominant ecological processes driving patterns of phytoplankton diversity. Finally, we investigate the ecological strategies selected by the hydrographic conditions of the front.

## Materials and methods

### Oceanographic context and sampling strategy

**Figure 1:**
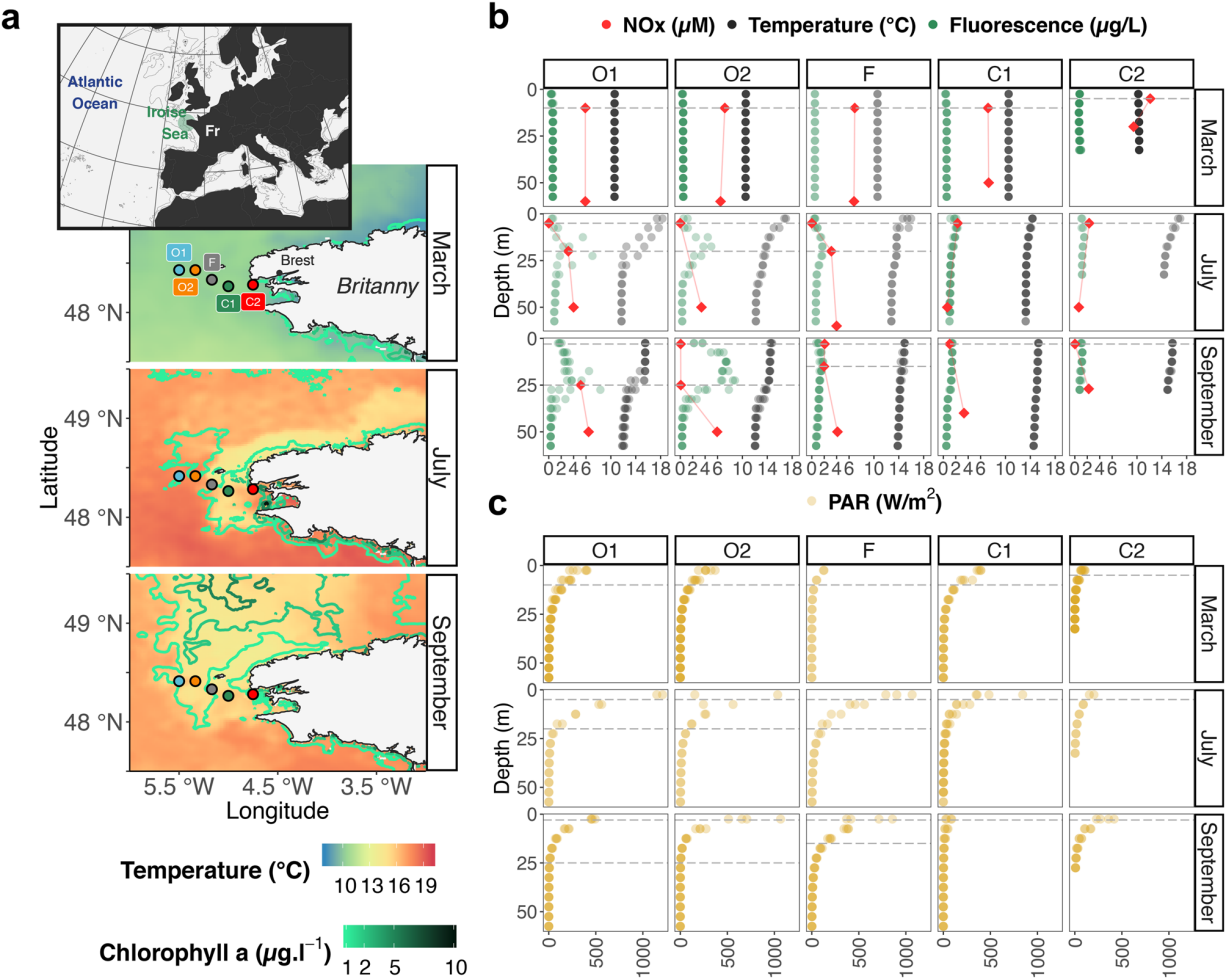
Map of the Iroise Sea and its hydrological conditions during our three sampling campaigns in 2015. On the map, the sampling stations (dots and names) are superimposed on the corresponding temperature (background color) and chlorophyll *a* (isoligns). Each map was assembled in R^50^ using raster images estimated with satellite data ( source: NASA Goddard Space Flight Center, Ocean Ecology Laboratory, Ocean Biology Processing Group. Moderate-resolution Imaging Spectroradiometer (MODIS) Aqua Ocean color Data; NASA OB. DAAC, Greenbelt, MD, USA. Accessed 2017/10/17). Vertical profiles of fluorescence (green, μg/L), temperature (black, °C), and PAR (yellow, W/m^2^) were measured on board with a CTD probe and a fluorescence sensor. Values of the down-cast were averaged every 5 m, variability comes from the triplicated casts. NOX (nitrate + nitrite, red, µM) were measured a posteriori from water sampled at three depths (a single time). Dashed horizontal lines represent the sampling depth for surface and, when present, DCM samples.

The Ushant tidal front forms in the Iroise Sea (Atlantic, Western France) and lasts from May to October^28^. Five stations distributed across the Ushant tidal front were sampled in our study; respectively from the open-ocean to the coast: O1, O2, F, C1 and C2 (Fig. 1a). The distance between stations was approximately 15 km, for a total transect of 58 km. The five stations were sampled three times during 2015, in early spring (10–12 March), early summer (1–3 July) and late summer (8–10 September). A sampling rosette equipped with Niskin bottles (10 L), a conductivity–temperature–depth probe (CTD) and a fluorescence sensor were used for profiling water temperature stratification, the chlorophyll *a* concentration and the Photosynthetically Active Radiation (PAR) over the water column (Fig. 1b,c). Water samples were collected at the surface (0–5 m) and, when present, at the deep chlorophyll maximum (DCM) identified by fluorescence profiling with the CTD. The water samples were triplicated by three repeated casts within 1 h and at the same geographic coordinates.

Seawater was processed with a sequential filtration approach in order to separate communities of micro-, nano- and pico-plankton (respectively > 10, 10–3 and 3–0.2 µm). Polycarbonate membrane filters of 47 mm in diameter were used for pore sizes of 10 and 3 µm, while polyether-sulfone sterivex were chosen for the pore size of 0.2 µm. For each sample, seawater was filtered until filter clogging, with volumes ranging from 2.7 to 5.6 L. The filters were frozen onboard in liquid nitrogen and later stored at − 80 °C until DNA extraction. An additional volume of water (0.5–1 L) was filtered through a 25 mm GF/F filter of 0.3 µm pore size, the filter was then placed in a cryotube and preserved at − 80 °C. These filters were later analyzed with High-Performance Liquid Chromatography analysis (Shimadzu) to quantify photosynthetic pigment concentration. These values were then used to calibrate the fluorescence sensor (Fig. 1b). Finally, 5–6 mL of seawater was filtered with a syringe holding a 25 mm GF/F filter of 0.3 µm pore size and preserved in ponyvials at − 20 °C. These last samples were used to estimate macronutrient concentrations using a Seal Analytical AA3 HR automatic analyzer following procedures described in reference^44^.

### DNA barcoding

DNA barcoding was used to infer eukaryotic phytoplankton diversity at high resolution. Genomic DNA was extracted from the polycarbonate filters using the DNA extraction kit NucleoSpin Plant II from (Macherey-Nagel, Hoerdt, France). The V4 region of the 18S rDNA gene, a marker gene conserved but highly variable across the taxa of the protistan community, was amplified using PCR, performed with a taq polymerase (Phusion High-Fidelity PCR Master Mix with GC Buffer) and eukaryote-specific primers^45^. Library preparation was performed following the protocols of the NucleoSpin Gel and PCR Clean-up kits (Macherey-Nagel, Hoerdt, France). Sequencing was performed at the Genotoul platform using Illumina Mi-Seq (http://get.genotoul.fr/).

Bioinformatics consisted of a sequence quality filter using built-in modules of USEARCH^46^, singletons removal as performed in reference^47^, and sequence taxonomic annotation with the PR^2^ database^48^. Using Swarm2^49^, similar variants of the amplified marker gene were clustered together to define Operational Taxonomic Unit (OTU), i.e. the species unit of DNA-barcoding. OTUs were annotated with the taxonomy of their most abundant variant. OTUs annotated to metazoan and pluri-cellular plants were removed. Further details about our DNA barcoding approach can be found in reference^43^, where bioinformatics have been performed on a larger dataset to improve error detection and OTUs clustering. Our dataset contains 33 060 OTUs accounting for 3.5 × 10^6^ reads. Many OTUs were annotated with the same taxonomy, corresponding to 1028 unique taxonomic references. The taxonomic composition of this dataset can be investigated in Supplementary Fig. S1.

### Eukaryotic phytoplankton diversity

All analyses were performed with the R software^50^ and the specific packages mentioned. Selection of OTUs representing eukaryotic phytoplankton was carried out by coupling our barcoding dataset with a trait database (available at www.seanoe.org/data/00405/51662/)^43^. The database is a custom trait annotation of the taxonomic references given to our OTUs (see DNA-barcoding section). After a literature review, we were able to identify 362 taxonomic references (from 1028) with an obligatory phototrophic lifestyle that represented eukaryotic phytoplankton^51^. We then created a sub-dataset of the 10,597 OTUs and 1.5 × 10^6^ reads that were annotated to these 362 taxonomic references (representing respectively 32% OTUs and 45% reads in the initial dataset). This sub-dataset was used to study patterns of phytoplankton diversity. Sampling quality was evaluated by rarefaction curves (read vs. OTU number; *rarecurve* function of R package “vegan”^52^; Supplementary Fig. S2), and an estimate of richness saturation was calculated using the R package “iNEXT”^53^.

Eukaryotic phytoplankton richness (number of OTUs per sample or alpha-diversity) was computed for each of our 184 distinct samples, corresponding to distinct season (3), station (5), depth (2 when the DCM was sampled), size-fraction (3) and triplicate. Phytoplankton richness was first analyzed using boxplots across seasons, stations and size-fractions. Secondly, replicates and depth samples were merged and phytoplankton richness was computed again to represent the total phytoplankton richness across seasons and stations (15 samples). All statistical tests were performed on the dataset made of 184 samples. Patterns of phytoplankton richness were tested with the Kruskal–Wallis test, a non-parametric one-way analysis of variance. Differences in community composition (occurrence and proportions of OTUs across samples) were tested with a Permutational Multivariate Analysis of Variance using the Bray–Curtis distance (PERMANOVA; *adonis* function of R package “vegan”)^52^.

The spatial structure of phytoplankton diversity was studied by focusing on the occurrence of OTUs in each station within a season. OTUs were categorized in ‘shared’, when they occurred in more than one station within a season, or to ‘specific to station X’, when occurring only in station X. Phytoplankton richness was also studied according to its abundance. Rank abundance curves were built by calculating the total read number of OTUs in each season. OTUs were then sorted according to their total read abundance. In these rank abundance curves, OTUs from all size-fractions are merged, we note that this approach blurs the potential disproportionate abundance of size-fractions in the environment and the compositional nature of our DNA-barcoding dataset.

### Community assembly of eukaryotic phytoplankton

To describe the spatio-temporal structure of eukaryotic phytoplankton, the number of OTUs shared between stations, within and across seasons, was computed with the R package “vegan” (*betadiver* function, metric α)^52^. To represent this metric, from now on called OTU-connectivity, we computed a network (with the R package “igraph”^54^) where nodes represent stations and edges width represent the number of OTUs shared between two stations. Surface and DCM samples were distinguished in this analysis. We tested if the sampling effect between seasons (different phytoplankton richness across seasons) affected OTU-connectivity by comparing the observed OTU-connectivity with the OTU-connectivity in a sub-dataset with a curated number of OTUs by season (see the experimental procedure in Supplementary Fig. S3). The correlation between the connectivity matrices (pairwise comparison of the number of OTUs shared between samples) of the original and the curated dataset were studied with the Spearman rank correlation. Further analyses of the connectivity network can be found in Supplementary Figure S4.

To quantify the ecological processes that constrained phytoplankton community patterns across the Ushant tidal front we followed the approach of reference^55^. The method has already been reviewed^37^, further details are given in Supplementary Figure S5. Briefly, the method is based on the comparison of observed community turnovers (shift in composition across samples), phylogenetic turnovers (shifts in composition weighted by phylogenetic similarity between taxa) and turnovers expected by chance (in null-models), in order to estimate whether the differences between pairs of communities are explained by Dispersal, Selection, or Ecological Drift^37^. The implicit hypothesis is that phylogeny is a proxy for niche divergence between taxa, so phylogenetic turnover can then be used to infer niche-based selection. This hypothesis has seldom been tested for phytoplankton^56^ and thus, before applying this method, the relationship between OTU-OTU phylogeny and trait-based distances was tested (Supplementary Fig. S5). The phylogenetic distance and tree were computed with R following reference^57^. The method comprises a sequence multiple-alignment (here eukaryotic phytoplankton V4 sequences of the 18S rDNA) and the computation of a neighbor-joining tree fitted to a GTR + G + I maximum likelihood tree. Phylogenetic turnover was estimated with the ß-Mean-Nearest-Taxon-Distance (ßMNTD) (*comdistnt* function of R package “Picante”^58^). Composition turnover was computed using Jaccard’s dissimilarity index^59^. 999 null-models were computed with built-in R functions, using random shuffling of the phylogenetic tree labels for null-models of phylogenetic turnover (ßMNTD)^55^ and using the Raup-Crick metric (RC) for null-models of composition turnover^59^. The ß-Nearest-Taxon-Index (ßNTI) was computed as the difference between the observed ßMNTD and the mean of the ßMNTD null-models. The inference of the dominant ecological process between a pair of sample was carried out following Refs.^55,60^; with |ßNTI| > 2 interpreted as a dominance of selection, |ßNTI| < 2 and |RC| > 0.95 interpreted as a dominance of dispersal and |ßNTI| < 2 and |RC| < 0.95 interpreted as a dominance of ecological drift. Negative values of ßNTI and RC represent communities that are more similar than expected by chance, selection and dispersal were thus considered ‘homogenous’ or ‘homogenizing’, at the contrary positive values represent communities where ‘variable selection’ and ‘dispersal limitation’ favor dissimilarity (higher than expected by chance). Compositionality, the phylogenetic uncertainty of a tree based on short amplicon size and potential biases in local richness are recognized limitations to this approach^55^. Therefore a safe approach was adopted by using non-weighted-metrics, testing the relationship between phylogeny and traits (Supplementary Fig. S5), and testing whether variable richness across seasons affected our interpretations (Supplementary Fig. S4).

To investigate the environmental variables forcing selective processes on phytoplankton communities, the significance of the difference in phylogenetic turnovers (ßMNTD) across environmental variables were tested with a PERMANOVA. Size fractions were distinguished in this analysis.

### Functional richness of eukaryotic phytoplankton

After investigating the patterns of taxonomic richness and composition of eukaryotic phytoplankton, we studied its functional richness with an innovative approach^61^. This method uses the estimation of species occurrence across samples and a trait-table describing these species, to compute functional richness. Using our trait database^43^, we generated a sub-dataset of 287 eukaryotic phytoplankton taxonomic references (out of 362) that were well annotated with the following morphological and trophic traits (12): SizeMin, SizeMax, Cell Cover, Cell Shape, Presence of Spicule, Cell Symmetry, Cell Polarity, Coloniality, Motility, Ingestion method, Symbiosis type and Resting Stage during the life cycle. These 287 taxonomic references constituted a sub-dataset of 7106 OTUs and 1.2 × 10^6^ reads (representing respectively 67% of OTUs and 80% of reads in the original phytoplankton dataset). To compare taxonomic and functional richness at large, OTUs occurrence was merged across seasons (3) and stations (5). Functional richness was then computed based on (a) this sub-dataset (7106 OTUs/15 samples) transformed in a presence–absence table, and (b) a trait table describing the 7106 OTUs of the sub-dataset (12 traits). Functional richness was computed with built-in R functions using Gower distance and Principal Coordinate Analysis (PCoA)^61^.

Finally, to investigate the ecological strategies of phytoplankton displayed across the Iroise Sea, we computed Gower’s Distance and the Complete Linkage’s method of Hierarchical Clustering^62^ on our trait table of 7106 OTUs and 12 traits (see this section). This approach identified 9 clusters of OTUs with similar trait combinations, thus representing ecological strategies (Supplementary Fig. S6). We focus on the distribution of these strategies in September by assuming that by the end of summer the accumulated selection processes might have narrowed the communities to few strategies.

## Results

### Oceanographic context

By analyzing physicochemical variables measured in the Iroise-Sea during a 2015 oceanographic campaign (Fig. 1a–c), we evidenced the Ushant tidal front in summer (July and September), with the most offshore stations (O1, O2) being stratified, as demonstrated by the temperature profiles, while temperature was more homogenous across depth in the coastal stations (C1, C2). As expected, the front was not yet established in March. Contrasting with the nutrient-replete waters of early-spring (March), the surface summer waters of the Iroise-Sea presented a marked nutrient depletion, at the exception of the waters at station F, and to a lesser extent in the coast at C1 and C2 (Fig. 1b). Phytoplankton production persisted at the DCM of the most offshore stations especially at station O2 in September, suggesting that phytoplankton at station O2 might benefit from the nutrient inputs and is close to the thermal front (Fig. 1b). However, lower nutrient (Fig. 1b) and light availability (PAR, Fig. 1c) might have constrained phytoplankton productivity in the rest of the stations. Station F presented a weaker stratification throughout summer and significant nutrient inputs at surface in September (Fig. 1b). This suggests that station F was the closest station to the front, although the front’s position probably oscillated between station F and O2 depending on tidal cycles and wind conditions.

### Phytoplankton community composition

**Figure 2:**
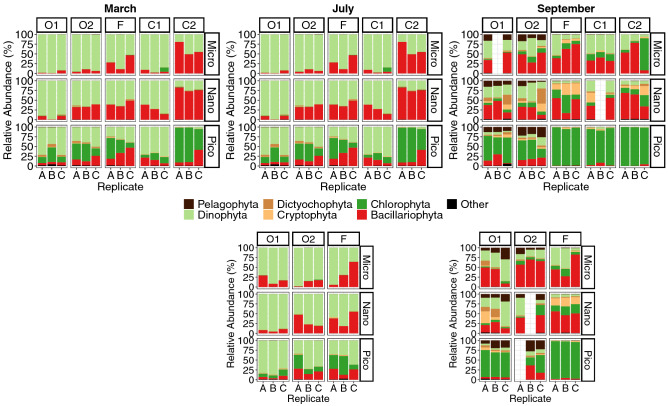
Proportions of eukaryotic phytoplankton taxa estimated by DNA-barcoding in the Iroise Sea in March, July and September 2015. Samples are organized by replicates, size-fractions, sampling stations, depths (surface and Deep Chlorophyll Maxima, DCM) and seasons. The relative abundance was calculated based on the number of reads of the OTUs corresponding to the shown phytoplankton taxa, ‘Other’ represents the proportion of taxa with a relative abundance < 10% over the dataset.

In this section, we first present the patterns of phytoplankton taxonomic groups across seasons (Fig. 2). The total phytoplankton read abundance (1.5 × 10^6^ reads) was dominated by *Bacillaryophyta* (i.e. diatoms, 36% of the total phytoplankton read abundance) and *Dinophyta* (i.e. dinoflagellates, 31%), that dominated micro-plankton. *Chlorophyta* (25%), *Cryptophyta* (5%) and *Dictyochophyta* (1%) were more abundant in the nano- and pico-plankton. Organisms from *Pelagophyta* (1.5%) were observed homogenously across all size fractions but appeared mostly in September in the offshore samples. As is typical for sequencing surveys^63^, DNA from organisms of specific size-fractions slightly contaminated other fractions due to cell-breakage (e.g. dictyochophytes, pelagophytes usually found in the pico-plankton or diatoms and dinoflagellates usually found more dominant in the micro-nano-plankton). The variability in community composition within triplicates was not significant (PERMANOVA, R^2^: 0.1 with 9999 permutations). More surprisingly, across stratified waters in July and September, no significant differences were found between the OTU compositions at surface and at the DCM (PERMANOVA, R^2^: 0.03 with 9999 permutations). Vertical mixing in the surface layers thus appeared sufficient to mix the communities from the DCM and the surface, making selection at different depth undetectable with our methodology.

In March, phytoplankton was dominated by diatoms in the micro- and nano-plankton (respectively above 75 and 40% of the total phytoplankton abundance), *Cryptophyta* appeared above 10% in both the nano- and pico-plankton, while *Chlorophyta* dominated pico-plankton (above 75% by sample, Fig. 2). During this period, the relative abundance of the phytoplankton taxa was homogenous across the Iroise Sea (Fig. 2). In July, the community was dominated by dinoflagellates in the higher size-fractions. However, diatoms remained abundant in the most coastal area (Fig. 2). Among pico-plankton, *Chlorophyta* was the most abundant taxa but dinoflagellates and diatoms also appeared consistently in this size-fraction. Pico-plankton was relatively homogenous over the Iroise Sea except for the most coastal station (C2) that showed a stronger domination of *Chlorophyta* (Fig. 2). In September, the spatial structuration was stronger across all size fractions. Micro- and nano-plankton were dominated by diatoms and to a lesser extent by dinoflagellates (Fig. 2). This season was mainly characterized by the presence of *Pelagophyta* and *Dictyochophyta* in the most offshore stations (above 10% and 3% in O1 and O2) with *Pelagophyta* appearing across all size fractions while *Dictyochophyta* appeared mostly in the nano-plankton (Fig. 2). The frontal and coastal stations (F, C1 and C2) showed noticeable abundances of *Cryptophyta* in the nano-plankton (above 10%). Across pico-plankton, stations were also divided into two groups, one with the coastal and frontal stations (F, C1 and C2) where *Chlorophyta* strongly dominated, and one with the offshore stations (O1 and O2) where *Chlorophyta*, *Pelagophyta*, diatoms and dinoflagellates were more evenly distributed (Fig. 2).

### Patterns of phytoplankton richness

In this section, we investigated patterns of eukaryotic phytoplankton richness using the total number of OTUs as a proxy. Rarefaction analyses indicated that reaching near saturation for the eukaryotic phytoplankton of the Iroise sea’s (13,659 OTUs) would require between 500 and 600 samples following our strategy (Supplementary Fig. S2). With 10,597 OTUs, our study did not saturated diversity but represents the diversity patterns of the most abundant share of the eukaryotic phytoplankton communities sampled. We detail the non-extrapolated richness retrieved in our original dataset.

**Figure 3:**
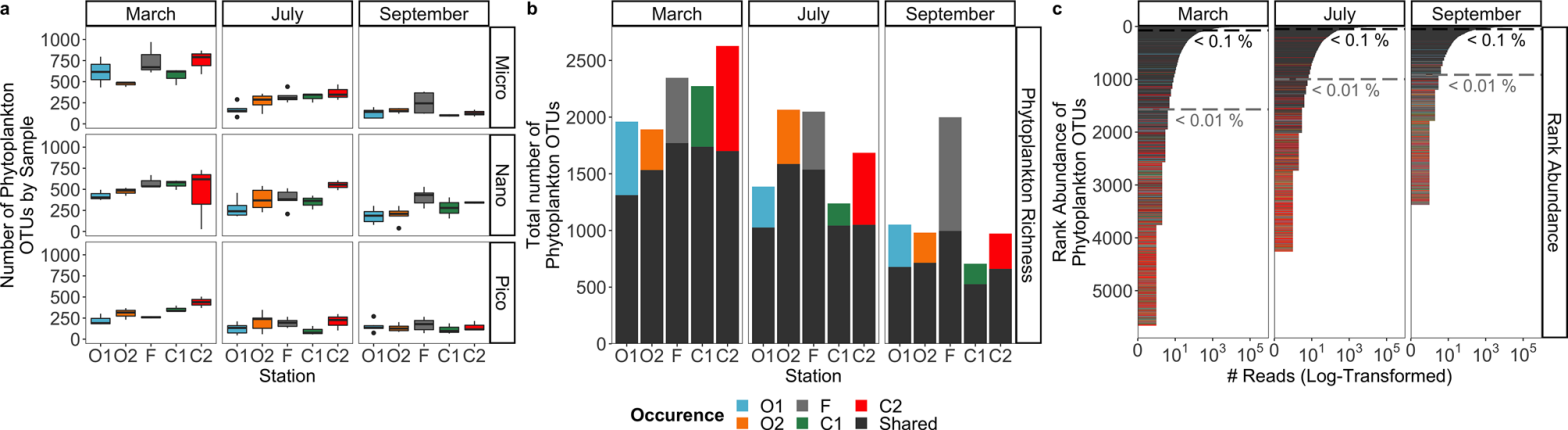
Eukaryotic phytoplankton richness patterns in the Iroise Sea in 2015. (**a**) Variability of phytoplankton richness (# of OTUs by samples) across our 184 samples. The figure represents the variability of phytoplankton richness (y-axis) across stations (x-axis), size-fractions (right panels) and seasons (top panels). The variability in these sections is represented by samples triplicated and sampled across two depths (when carried out). (**b**) Total merged phytoplankton richness over our 5 stations and 3 seasons (size of barplots, 15 samples). The color code in each barplot represents two categories of occurrence, the OTUs that are ‘shared’ (in dark gray) by at least two stations in a same season, and the OTUs ‘specific to station X’ within a season (see the respective colors in the legend). This color legend is shared with (**c**). (**c**) Rank abundance curves of all eukaryotic phytoplankton OTUs found in each season. Each OTU is represented by a bar in the graphic, the color of the bar corresponds to the same categories of occurrence as (**b**). The number of reads of OTUs was log-transformed for the sake of presentation. The dotted lines separate OTUs across abundance thresholds, the abundant OTUs (> 0.1% of the total read number of phytoplankton OTUs by season), the low abundant OTUs (0.1–0.01%) and the rare OTUs (< 0.01%).

The variability of eukaryotic phytoplankton richness (i.e. the number of phytoplankton OTUs in a sample) in the Iroise Sea throughout 2015 is presented in Fig. 3a (for all 184 samples considered independently). Phytoplankton richness was significantly higher in micro- and nano-plankton than in the pico-plankton (*P* < 0.0001, Kruskal–Wallis test). Micro- and nano-plankton presented the highest richness (maxima of 970 and 729 OTUs respectively) whereas pico-plankton presented lower values (maximum of 504 OTUs). Phytoplankton richness also declined significantly along seasons (*P* < 0.0001, Kruskal–Wallis test; Fig. 3a) and this decline was strongest for the micro- (maxima of 970, 467 and 381 respectively in March, July and September) than for nano- and pico-plankton (maxima of 729 and 504 OTUs in March, to 529 and 270 OTUs in September, respectively for nano- and pico-plankton). Among stations that presented DCMs and in agreement with results on community composition, no significant difference existed in phytoplankton richness between DCM and surface samples (*P* = 0.1, Kruskal–Wallis test).

To better represent the spatio-temporal structure of phytoplankton richness, OTU counts from replicates, size-fractions and depths were merged (15 samples; Fig. 3b). As expected, richness increased across stations and seasons (Fig. 3b). This increase was more influenced by merging replicates and size-fractions than by merging samples from different depths, in accordance with tests showing no significant difference in phytoplankton composition (PERMANOVA, R^2^: 0.03 with 9999 permutations) and richness (*P* = 0.1, Kruskal–Wallis test) between superficial and DCM samples. Along seasons and parallel to a depletion in nutrients, the total phytoplankton richness declined, with maxima of 2628, 2066 and 1999, respectively in March, July and September (Fig. 3b). However, across stations there existed discrepancies to this pattern. In July, the stations nearest to the front (F and O2) were richer than the others (above 2000 phytoplankton OTUs in comparison with values lower than 1700 OTUs in other stations), while in September, station F showed the highest phytoplankton richness (1999 OTUs in comparisons with < 1100 OTUs elsewhere). Consequently, richness was significantly higher at station F over summer (*P* < 0.005, Kruskal–Wallis test based on the complete dataset of 184 samples), highlighting that the front represented a hotspot of phytoplankton diversity.

To investigate the composition of this hotspot, OTUs were sorted according to their occurrence in each station by season (Fig. 3b). Across July and September, when the front was established, Station F showed a significantly higher richness of ‘shared’ OTUs (*P* < 0.01, Kruskal–Wallis test based on the summer-only dataset, 140 samples). When cumulated, its proportion of ‘specific’ OTUs reached 50% of phytoplankton richness in September (station F: 997 ‘Specific’ + 1002 ‘Shared’ OTUs), the highest value across stations and seasons (Fig. 3b). Next, the ‘shared’ OTUs constituted the larger part of the ‘abundant’ community (OTUs > 0.1% of the total read number of phytoplankton OTUs by season; Fig. 3c). ‘Specific’ OTUs were present in the ‘low abundance’ community (0.1–0.01%) and numerous in the ‘rare’ community (< 0.01%; Fig. 3c). This indicated that the most abundant species had a larger dispersal (and detection potential), while the distribution of rarer taxa was sparser and spatially structured. The numerous ‘specific’ OTUs found at Station F in September thus had low abundances. The rank abundances also highlighted that the shrinking of phytoplankton richness across seasons was mostly due to the rare community (with 4092, 3262 and 2462 of rare OTUs respectively in March, July and September; Fig. 3c).

### Phytoplankton turnovers, dominant ecological processes and environmental drivers

**Figure 4:**
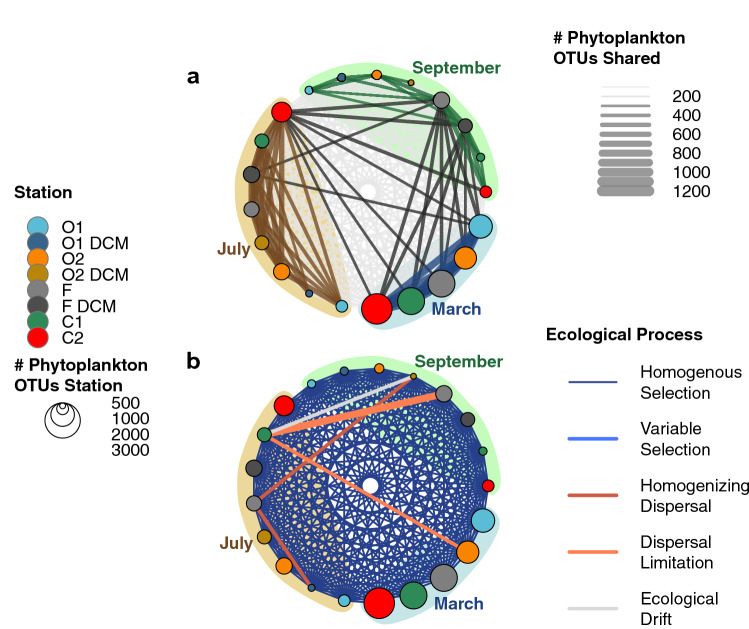
Spatiotemporal patterns of diversity and the dominant ecological processes driving eukaryotic phytoplankton communities sampled in the Iroise Sea in March, July and September 2015 across our 5 stations and depth (at surface and DCM). (**a**) OTU-connectivity network, the node size represents the number of OTUs in each station (see node color) and each season; the edge width represents the number of OTUs shared between stations; the edge color represents: low connectivity (light grey in the background, < 300 OTUs shared), intra-seasonal connectivity (colored) and cross-seasonal connectivity (black). (**b**) Dominant ecological processes explaining the pairwise differences between phytoplankton communities sampled in the Iroise Sea, the edge color represent the dominant ecological processes between the communities of each station as estimated in the approach of reference^55^. On the figure, we put emphasis on processes different from Homogenous Selection, the process dominating 5541 out of the 5551 pairwise community comparisons in our dataset. The edge width linking ‘C1 in July’ and ‘F in September’ was doubled because an ecological process other than Homogenous Selection appeared more than once (across replicates of the micro-plankton). All networks were plotted using the R package “igraph”^54^.

As a preliminary step to this section, we tested if OTU-connectivity (Fig. 4a) was influenced by the decreasing number of OTUs by season observed in Fig. 3, a potential bias in our analysis. The observed diversity patterns were conserved in a dataset with a curated number of OTUs by season (Spearman Rank Correlation of 0.99; Supplementary Fig. S3), indicating that OTU-connectivity was not influenced by the varying number of OTUs retrieved in each season, we thus detail the observed patterns. First, OTU-connectivity was significantly higher within seasons than across seasons (Fig. 4a; Supplementary Fig. S4, *P* < 0.001, Kruskal–Wallis test), illustrating a seasonal renewal of the phytoplankton community. Secondly, the connectivity between stations within a season significantly decreased from early spring to late summer (Fig. 4a; Supplementary Fig. S4, *P* < 0.001, Kruskal–Wallis test), highlighting the progressive separation of communities across stations. Finally, cross-seasonal connectivity indicated significant patterns: (1) in March, all stations were rich and phytoplankton OTUs were widespread (high connectivity intra-season), (2) in July, some OTUs found in March still occurred in the coastal and the frontal stations (C2 and F, see the cross-seasonal links in between March and July), indicating a strong renewal of the phytoplankton community elsewhere, and 3) in September, OTUs sustained from March and July were found in a greater extent at the front (cross-seasonal link in between July and September). As a consequence, OTU-connectivity at the frontal station was higher than at other stations, both within (Fig. 4a; Supplementary Fig. S4, *P* < 0.001, Kruskal–Wallis test) and across seasons (Fig. 4a; Supplementary Fig. S4, *P* < 0.001, Kruskal–Wallis test).

The null-model approach based on phylogenetic and compositional turnovers supposed that eukaryotic phytoplankton was strongly affected by selection (Fig. 4b). Out of 5551 pairwise community comparisons, 5545 were dominated by Homogenous Selection, 3 by Dispersal Limitation, 2 by Homogenizing Dispersal and 1 non-dominated (supposedly representing Ecological Drift). The domination of Homogenous Selection supposes that the OTU-composition across stations remained very similar throughout our sampling (as evidenced by OTU-connectivity, Fig. 4a), and followed the same selection process over time. The pairwise community comparisons where selection was relaxed (i.e. where |ßNTI| < 2) were all (6) within the micro-plankton and 4 involved the frontal station. Non-selective pairwise comparisons also evidenced the increasing structuration of the micro-phytoplankton across the front in summer (Fig. 4b), with dispersal limitation reducing the similarity between the offshore and the coast (O2 in March, C1 in July), and between the coast and the frontal station (C1 in July, F in September). However, the frontal station, richer in OTUs, also contributed through dispersal to the community composition on the offshore side of the front (O1 in July, O2 in September, F in July).

**Figure 5:**
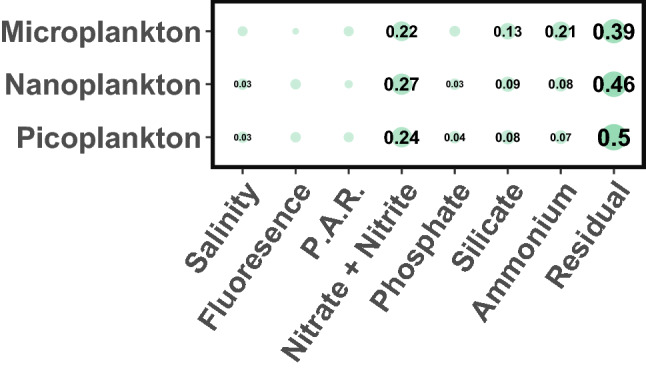
Permutational multivariate analysis of variance (PERMANOVA) between the phylogenetic community turnovers of eukaryotic phytoplankton (ß-Mean Nearest Taxon Distance) and hydrographic variables measured in the Iroise Sea. The value of the R^2^ of the PERMANOVA is displayed when the p value between the two variables was < 0.05. The analysis was repeated for each size-fraction.

The factors driving phytoplankton selection were investigated using PERMANOVA between phylogenetic turnovers (ßMNTD) and the environmental factors during our cruises. Two PERMANOVAs were computed, both showed that our environmental factors explained between 50 and 62% (across PERMANOVAs and size-fractions) of the turnovers variance, supposing that the rest of the variance was explained by unmeasured factors. The first PERMANOVA highlighted a strong correlation between temperature and phytoplankton phylogenetic turnovers (R^2^ for the micro: 0.24, nano: 0.27, picoplankton: 0.28 with p values of 0.001); in agreement with our sampling strategy targeting a seasonal increase in temperature and a thermal front (Fig. 1). To elaborate on this result, a second PERMANOVA was computed without temperature (Fig. 5). In this analysis, nutrients correlated with phytoplankton turnovers, most notably nitrate + nitrite, globally decreasing across seasons except at the front (R^2^ for the micro: 0.22, nano: 0.27, picoplankton: 0.24 with p values of 0.001), ammonium, globally increasing with the summer remineralization of the spring bloom (R^2^ for the micro: 0.21, nano: 0.08, picoplankton: 0.07 with p values of 0.001), but also to a smaller extent silicate, mostly impacting diatoms’ growth (R^2^ for the micro: 0.13, nano: 0.09, picoplankton: 0.08 with p values of 0.001). This analysis illustrates the strong selective effect of water temperature and nutrient availability on the phytoplankton community of the Iroise Sea.

### Phytoplankton functional diversity

Using a trait database adapted to our DNA barcoding dataset, we first tested if the hotspot of phytoplankton diversity also corresponded to a higher number of ecological strategies. Secondly, we investigated if the observed selection pressures resulted in distinct sets of ecological strategies across the front by the end of summer.

**Figure 6:**
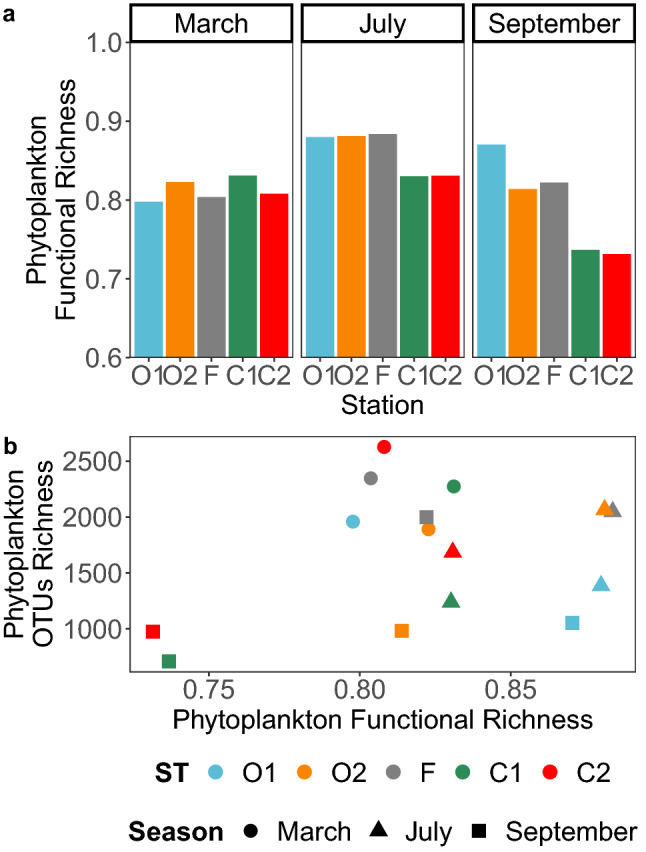
Patterns of eukaryotic phytoplankton functional richness in the Iroise Sea in 2015. (**a**) Functional richness was computed based on the phytoplankton OTUs (7106) present in each station (3) and each season (3) that were annotated with 12 biological traits. The trait annotation was carried out during a previous literature survey^43^, and the metric was calculated by following reference^61^. (**b**) Pairwise comparisons of phytoplankton functional richness and taxonomic richness (OTUs) in our dataset.

Functional richness, ranging between 0 and 1, was high (> 0.7) supposing that all stations and seasons presented several distinct phytoplankton strategies (Fig. 6a). Contrary to taxonomic richness, the functional richness of phytoplankton did not show its highest values in March (between 0.79 and 0.83) but in July and September (ranging between 0.87 and 0.88). These high values were observed in the offshore and frontal area in July (O1, O2, F) but only at the most offshore station O1 in September (Fig. 6a). Despite a taxonomic richness twice lower than observed at the frontal station (Fig. 3), the most offshore area presented equivalent or higher functional richness than the frontal station. This suggests that the hotspot of diversity found at the front in summer presented a high number of taxa with similar ecological strategies, i.e. functional redundancy. Overall functional redundancy also explained the absence of correlation between the taxonomic and functional richness of eukaryotic phytoplankton (Fig. 6b).

**Figure 7:**
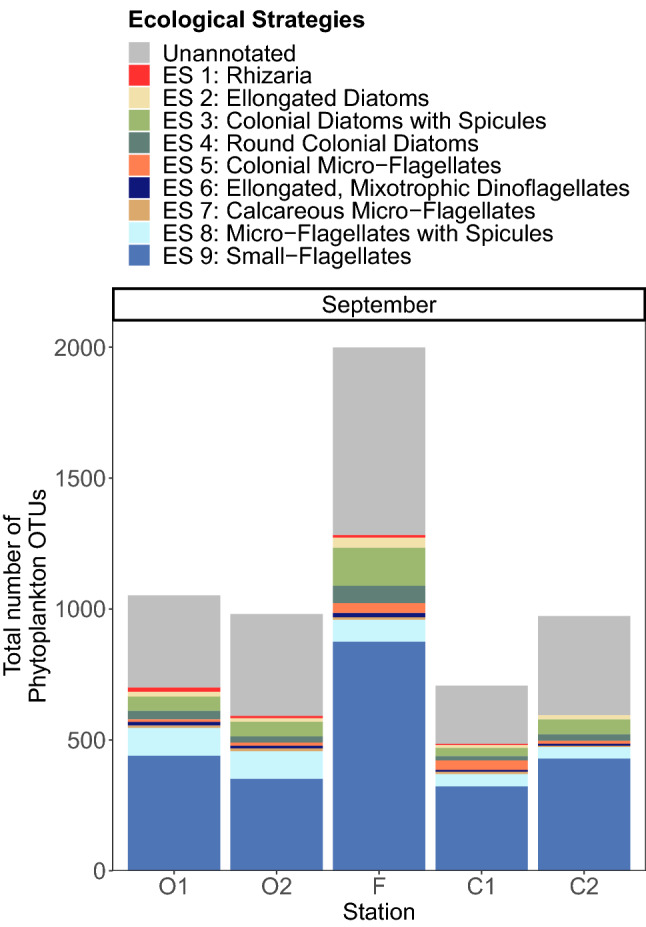
Phytoplankton richness within 9 ecological strategies of phytoplankton displayed across five sampling stations of the Iroise Sea in September 2015. Phytoplankton OTUs were sorted into distinct ecological strategies (see legend) represented by trait combinations (conserved within the groups) and when possible annotated with taxonomic affiliations. The gray area of the barplots represents the richness of the OTUs that could not be annotated with traits or with an ecological strategy.

By studying the distribution of 9 distinct ecological strategies of phytoplankton (Supplementary Fig. S6) across the front in September, we evidenced that the front did not select a specific ecological strategy (Fig. 7), despite a higher number of OTUs. Only the Rhizarians (ES 1); a very distinctive ecological strategy composed of OTUs with silica or strontium sulfate covers, spicules, able of phagotrophy and often carrying photosymbionts; showed a cross-frontal pattern with an increase of the OTUs number towards the open ocean (Fig. 7), despite overall low abundances in our dataset.

## Discussion

### Phytoplankton community composition

The Iroise Sea presented various environmental conditions that influenced the proportions of phytoplankton taxa due to differences in environmental niche and fitness.

Diatoms dominated the larger size-fractions (micro- and nano-phytoplankton) during early-spring (March) when light only started to become available in the nutrient-rich and mixed Iroise Sea. This supports evidence that diatoms are good competitors under high nutrient concentration^64^. The summer conditions represented nutrient-depleted and stratified waters and were dominated by dinoflagellates. Dinoflagellates have indeed better competitive abilities in such conditions (e.g. swimming to persist in the euphotic zone when mixing is low, or mixotrophic capabilities to compensate for low nutrient concentration)^64–66^. In summer, the coastal and frontal waters were markedly less depleted (NOx ~ 2 µM) than the open-ocean waters, these conditions thus helped to maintain a diatom community mixed with dinoflagellates. The smaller Cryptophytes occurred in the nano-phytoplankton and showed ubiquitous distribution in early-spring (March) as well as high proportions at the coast in September. Because of their size, Cryptophytes have lower light requirements^67^ and tend to favor coastal ecosystems^63^, which could explain their occurrence in March and restraint at the coast in September when the rest of the Iroise Sea presented more oceanic influences (Fig. 1). Finally, the pico-phytoplankton was dominated by Chlorophytes, as is typical in coastal ecosystems^63^. Due to their tiny size^68^, Chlorophytes have even lower requirements which allow them to grow in winter or in DCMs where the growth of large micro-phytoplankton organisms is greatly lowered^67^. In the Iroise Sea, light limitation also occurs in the shallower coastal area during summer due to vertical mixing and turbidity^69,70^, which again favored Chlorophytes in these areas (Fig. 2). In contrast, the lower proportion of Chlorophytes at the DCM of offshore stations, another light-limited environment, could be explained by the competition with other pico-eukaryotes. Indeed, both Pelagogphytes^71^ and Dictyochophytes^72^, observed in the most offshore areas, have a marked preference for deeper and stratified water masses.

Our environmental analysis (Fig. 5) partly agreed with these theoretical interpretations^64,65,68^. Nutrients and temperature were shown to be major drivers of phytoplankton selection (Fig. 5), but light availability (estimated with P.A.R.) did not seem to affect phytoplankton community patterns. This discrepancy can be explained by the fact that our measurements represent only snapshots of the environment and do not inform on its history (e.g. the past and daily exposure to light). Estimation of grazing pressure is also missing in our study but is likely to influence phytoplankton community patterns^73^. Nevertheless, our DNA barcoding approach detailed communities of eukaryotic phytoplankton under strong selective pressure, and this with a high taxonomic resolution illustrated by the addition of less-studied organisms like dictyochophytes or pelagophytes.

### The formation of a phytoplankton diversity hotspot

Selection processes also affected eukaryotic phytoplankton richness. Here, we detail the ecological processes leading to these patterns by focusing on taxonomic richness and turnovers of eukaryotic phytoplankton.

First, phytoplankton richness decreased significantly across seasons. Although our turnover approach would benefit from a higher resolution (spatial, temporal, phylogenetic and in the estimation of taxa’s abundance), it provided us with arguments to say that homogenous selection was the dominant ecological process affecting phytoplankton communities (Fig. 4b). This process dominates when communities appear in the same selective environment and when selection is strong^60^. In the geographic scale of our study (58 km), the Iroise Sea thus represented a homogenous pool of phytoplankton OTUs undergoing similar selection pressures. Additional analyses highlighted that temperature gradients and nutrient availability were important drivers of selection (Fig. 5), as is customary for phytoplankton taxa^64,65^. Under such continuous selection, the competition between taxa is predicted to increase, fewer taxa with better abilities under selection will survive^38,39^, resulting in the overall shrinking of phytoplankton richness that we observed in the Iroise Sea towards the end of summer (Fig. 3). Nutrient-driven selection probably explained why the shrinking of phytoplankton richness appeared mostly in the larger size-fractions, as the smaller organisms are usually more resistant to nutrient depletion^67^.

In contrast with the rest of the Iroise Sea, the frontal area maintained a higher phytoplankton richness across summer (Fig. 3), resulting in a phytoplankton diversity hotspot. Studying phytoplankton turnovers, we evidenced that selection was more relaxed over the front (Fig. 4b). Indeed, as in other fronts in summer, tidal mixing allows the upwelling of cold and nutrient-rich waters up to the surface of the Ushant tidal front^31^ (Fig. 1b). These nutrient inputs could have lowered selection and the resulting competitive exclusion, thus favoring higher richness. However, under continuous, or undisturbed, nutrient conditions, competitive exclusion between phytoplankton taxa is also predicted to increase^73^. Over the Ushant tidal front, disturbances originating from the spring/neap tide cycle and wind-induced perturbations are known to affect nutrients and light availability for surface phytoplankton^34,35^. In a mechanism reminiscent of the Intermediate Disturbance Hypothesis^74,75^, we speculate that the coupled cycles of tide-induced disturbances and nutrient inputs lowered competition locally, which helped to maintain a phytoplankton diversity hotspot over the front. It must be emphasized that these disturbances act at a short time scale, tangible with our yearly survey, but cannot prevent competitive exclusion and extinction in the long term^76^.

Focusing on the origin and connectivity of OTUs in the Iroise Sea, we evidenced that the front harbored a higher proportion of phytoplankton OTUs from surrounding areas (Figs. 3b, 4a). This suggests that the front also acted as an ecotone between the coastal and offshore area, allowing the local mingling of OTUs from separated water masses. This ecotone was probably favored by the displacements of the front that allowed the mixing of water masses and communities from neighbor areas^77^. Although dispersal is a common feature of phytoplankton diversity patterns^8^ (especially across large^12,78^ or smaller oceanic fronts^13,21^), the existence of phytoplankton ecotones is seldom reported^79^. Two scenarios can be envisioned for the sustaining of an ecotone at the front: (1) OTUs (quiescent or growing) migrated recently in detectable proportions, or (2) OTUs have migrated earlier but have been sustained locally in detectable proportions. Both scenarios were evidenced, as the frontal station showed a higher OTU-connectivity: (1) within seasons, supposing recent migration, and (2) across seasons, supposing earlier migration of sustained OTUs (Fig. 4a; Supplementary Fig. S4). Water mixing was thus crucial in bringing together OTUs from separated areas, while the relaxation of selection favored the sustaining of OTUs over the front^21^.

Finally, by studying the patterns of rare and abundant phytoplankton OTUs in late summer, we evidenced that 50% of the diversity hotspot was made-up of OTUs specific to this station (Fig. 3b), most of which were shown to be rare (Fig. 3c). The maintaining of rare OTUs at the front contrasted with the shrinking of the rare phytoplankton richness across seasons (Fig. 3c). Hence, our approach seems to support the results of a recent theoretical study which hypothesize that reduction of competition in the vicinity of fronts mostly promotes rare protistan diversity^80^. Overall, the tidal front thus shaped a hotspot of eukaryotic phytoplankton diversity by mixing the abundant OTUs of neighboring areas and by lowering competitive exclusion, which sustained a higher number of rarer phytoplankton OTUs.

### Hindsight from a trait approach

With a trait approach, we studied the ecological strategies that were selected in the Iroise Sea throughout summer. The principal goals were to test: (1) the existence of ecological strategies selected by the Ushant tidal front, and (2) if the hotspot of phytoplankton taxonomic diversity was also translated in a high richness of ecological strategies.

In September, after months of selection, the successful ecological strategies found at the front were not significantly distinct from those found in other areas (Fig. 7). This result invalidates the hypothesis that a coastal tidal front could select one or more specific strategies^81^, e.g. a strategy adapted to the variability of the front’s hydrographic features^23,34^. The lag between phytoplankton’s generation time (from one to a few days^67,82^) and the period of dominant disturbances at the front (14 days for the spring/neap cycle^23,35^) probably explains the lack of an emergent adapted strategy to the front.

The frontal area presented high phytoplankton taxonomic richness that did not translate into a higher functional richness by the end of summer (Fig. 6). This result supposes a high functional redundancy in the hotspot of diversity^83^. In a previous study of lake phytoplankton^84^, a decrease in competition also led to higher species richness with however a small variance in traits. The authors supposed that only a share of the present ecological strategies (or trait space in reference^84^) was affected by the decrease in competition. As a consequence, taxonomic richness was sustained only within these selected strategies leading to higher redundancy. However, we ruled this mechanism out, as the front did not appear to favor a specific strategy (Fig. 7). Our results are perhaps more reminiscent of a study on phytoplankton from coastal lagoons, where authors found that selection-induced competitive exclusion occurred primarily within functional groups^85^. Indeed, in areas other than the front all ecological strategies were present in similar proportions, but intra-strategy richness shrunk.

Our trait approach remains flawed by the lack of biological knowledge on most marine protists, which limits the number of OTUs and traits investigated, as well as the potential ecological interpretations^43^. As an example, it is impossible to rule out the fact that other types of ecological strategies (e.g. based on traits related to uptake or thermal preferences not included in our trait base) could be specific to the front^43^. Weighted functional metrics (weighting by the abundance or biomass of the taxa) are also likely to put more emphasis on the selected taxa which would likely yield distinct patterns of functional diversity^83^. In our non-weighted approach (due to the use of DNA-barcoding), the patterns of functional richness were indeed conditioned by the presence of Rhizarians, which represent a very distinct ecological strategy but were not among the most abundant taxa. We advocate for the development of further trait approaches in order to gain better insights on the interplay between phytoplankton and their ecosystem.

## Conclusion

Using oceanographic measurements, DNA barcoding and a trait approach we detailed the setting of a eukaryotic phytoplankton diversity hotspot over a coastal tidal front. Our results reach a consensus with previous theoretical approaches concerning the combined effect of dispersal and selection processes on the sustaining of phytoplankton diversity at the meso- and submeso-scale^14,18,19^. We generalized and confirmed previous results from few phytoplankton taxa to the whole eukaryotic phytoplankton community^21^, and we detail how these processes seem to affect taxa differently according to their abundance. We detail processes leading to functional redundancy in the hotspot of phytoplankton diversity, however we cannot rule out that the frontal area selects species according to non-annotated traits, most notably traits involved in temperature and nutrient-based selection. Through its cascading effects on zooplankton, fish and fisheries^23,86^, this phytoplankton diversity hotspot is essential to the large and productive food-web of the Iroise Sea^33,70,87^. A higher frequency in the sampling of phytoplankton communities, a better approximation of the variables structuring them (e.g. light availability, grazing pressure) and further hindsight on the history of water masses are still required to calibrate models at the regional scale^20^. Understanding and predicting the interdependent relationship between oceanic processes and the organisms that depend on them remains challenging but crucial, especially in the face of climate change and its predicted impact on oceanic ecosystems.

## Supplementary Information

Supplementary Information

## Data Availability

Our sequencing dataset and associated metadata are available at: https://sextant.ifremer.fr/record/16bc16ef-588a-47e2-803e-03b4acb85dca/. The trait database of marine protist is accessible at: https://doi.org/10.17882/51662.
