# Supplementary material for: Phytoplankton taxonomic and functional diversity patterns across a coastal tidal front
Source: Sci Rep. 2021 Jan 29;11:2682. doi: 10.1038/s41598-021-82071-0 (PMC7846791; doi:10.1038/s41598-021-82071-0)
Supplement: Supplementary file 1 — Supplementary Information [file 41598_2021_82071_MOESM1_ESM.pdf]

Supplementary Material and Figures

Title: Phytoplankton taxonomic and functional diversity patterns across a coastal tidal front

Authors and affiliations: Pierre Ramond*[1,2,3], Raffaele Siano[2], Sophie Schmitt[2], Colomban de Vargas[1,4], Louis Marié[5], Laurent Memery[5], Marc Sourisseau[2]

[1] Sorbonne Université, CNRS - UMR7144 - Station Biologique de Roscoff, Place Georges Teissier, 29688 Roscoff, France

[2] Ifremer - Centre de Brest, DYNECO/Pelagos, Technopôle Brest Iroise, 29280 Plouzané, France

[3] NIOZ - Royal Netherlands Institute for Sea Research and Utrecht University, Department of Marine Microbiology and Biogeochemistry, Den Burg, The Netherlands

[4] Research Federation for the Study of Global Ocean Systems Ecology and Evolution, FR2022/GOSEE, 3 rue Michel-Ange, 75016 Paris, France

[5] Laboratoire d'Océanographie Physique et Spatiale, UMR 6523 CNRS-IFREMER-IRD-UBO, Brest, France.

Corresponding Author:

Pierre Ramond

Phone: +33 6 87 44 15 59, Email: pierre.ramond@ifremer.fr

Figure S1: Distribution of the distinct protistan taxa estimated by metabarcoding in the Iroise Sea in March, July and September 2015. Samples are organized by replicates, size-fractions, sampling stations (from the open-ocean to the coast, left to right), depth and season. Relative abundance was calculated based on the number of reads of OTUs corresponding to the shown taxa, ‘Other’ represented the read number of taxonomic ranks with a relative abundance < 10%, ‘Undetermined’ represented the read number of OTUs with a low taxonomic level.

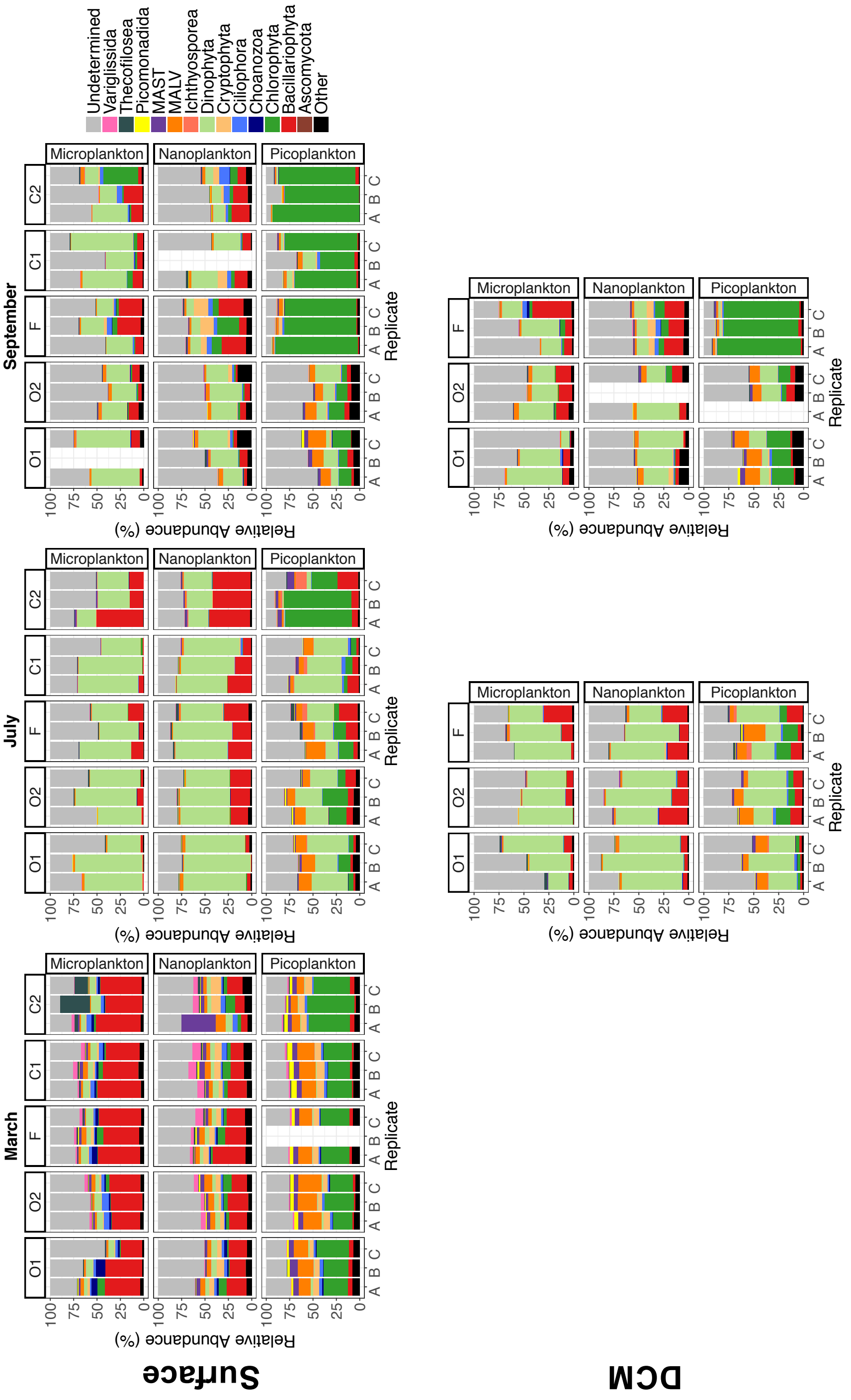

The total read abundance in our dataset was dominated by OTUs with well annotated taxonomy (66% at least annotated to the family level). Abundant taxa were: Dinophyta (i.e. dinoflagellates, 25% of the total read abundance), Bacillaryophyta (i.e. Diatoms, 14%), Thecofilosea (2%), Cryptophyta (2%) and Ciliophora (i.e. ciliates, 1.5%) that dominated micro- and nano-plankton; while Chlorophyta (10%) and Marine Alveolates and Stramenopiles (MALV: 5% and MAST: 2%) dominated pico-plankton. Due to cells-breakage, DNA from organisms with a usual cell diameter larger than 10 µm, was found across nano- and pico-plankton. Among replicates, the same clades dominated but there existed small changes in the relative abundance due to replicate corresponding to repeated rosette dives and casts on the same geographic location. Across stratified waters in July and September, no significant difference was found between the OTU composition at surface and at the DCM based on a Permutational Multivariate Analysis of Variance using the Bray-Curtis distance (PERMANOVA, R^2: 0.03 with 9999 permutations). Across OTUs annotated with trophic preferences (66% of total read number), constitutive phototrophs were dominating (42% of total read number) but heterotrophs were significantly present (13%). The rest (44% of total read) was composed of OTUs annotated at a low taxonomic level (e.g. Order, Class, Division), for which functional traits could not be collected.

Figure S2: Rarefaction curves and richness saturation analyses of all marine protists (A, B) and all eukaryotic phytoplankton OTUs (C, D, E) in our dataset of the Iroise Sea in 2015. The curves were constructed by cumulating the samples of each season (A, C) and of each size-fraction (B, D) independently. The sequencing depth is represented by the number of reads in relation to the species richness as the number OTUs. The function *rarecurve*() [function of "vegan" [1]] samples an increasing number of reads with a rate of 100 000 reads/sample and without replacement. The total number of eukaryotic phytoplankton OTUs in the Iroise Sea (E) was estimated based on our dataset and using the extrapolation method of the iNEXT package [2].

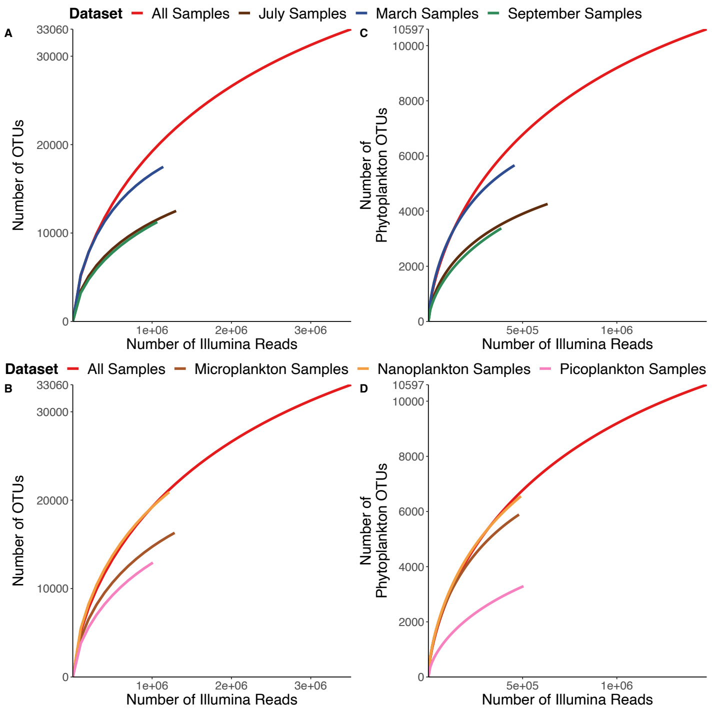

- None of the rarefaction curves reach an asymptote supposing that biodiversity was not saturated during our campaigns. We nevertheless retrieved 33 060 protistan OTUs and 10 597 OTUs. More sampling effort should increase these numbers.
- The sampling campaign in March retrieved more protistan OTUs compared to July and September (17 000 compared to 12 and 10 000) despite a similar sequencing depth (between 1 and 1.2 $x10^6$ reads).
- The sampling campaign in March retrieved more phytoplankton OTUs compared to July and September (5700 compared to 4300 and 3400) despite a similar sequencing depth (between 0.4 and 0.6 $x10^6$ reads).

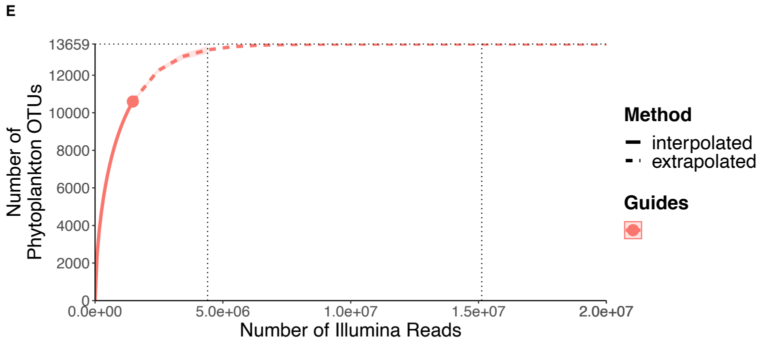

Based on our dataset, the iNEXT method allowed us to estimate that the total eukaryotic phytoplankton OTU-richness of the Iroise Sea would be 13.659 (Figure S2E). To reach this number of OTUs would require approximately 1.5 x 10^7 Illumina reads. To reach near asymptotic richness saturation would require ~4,5 x 10^6 Illumina reads. In our dataset, we have sequenced 1.5 x 10^6 Illumina reads with 184 samples, thus to reach near richness saturation would require (184*4.5)/1.5 = ~552 samples (three times our dataset).

Figure S3: OTU-Connectivity patterns of eukaryotic phytoplankton OTUs in a dataset with a curated number of OTUs by season and comparison to the original dataset of the Iroise Sea in 2015.

A) Normalisation processes to get a similar number of phytoplankton OTUs by season (left) and the effect of the removal on the original dataset (right), method inspired by [3]; B) Results of the normalisation processes on the OTUs connectivity across stations and seasons; C) Pairwise comparison of the connectivity matrices of the original dataset and the curated one.

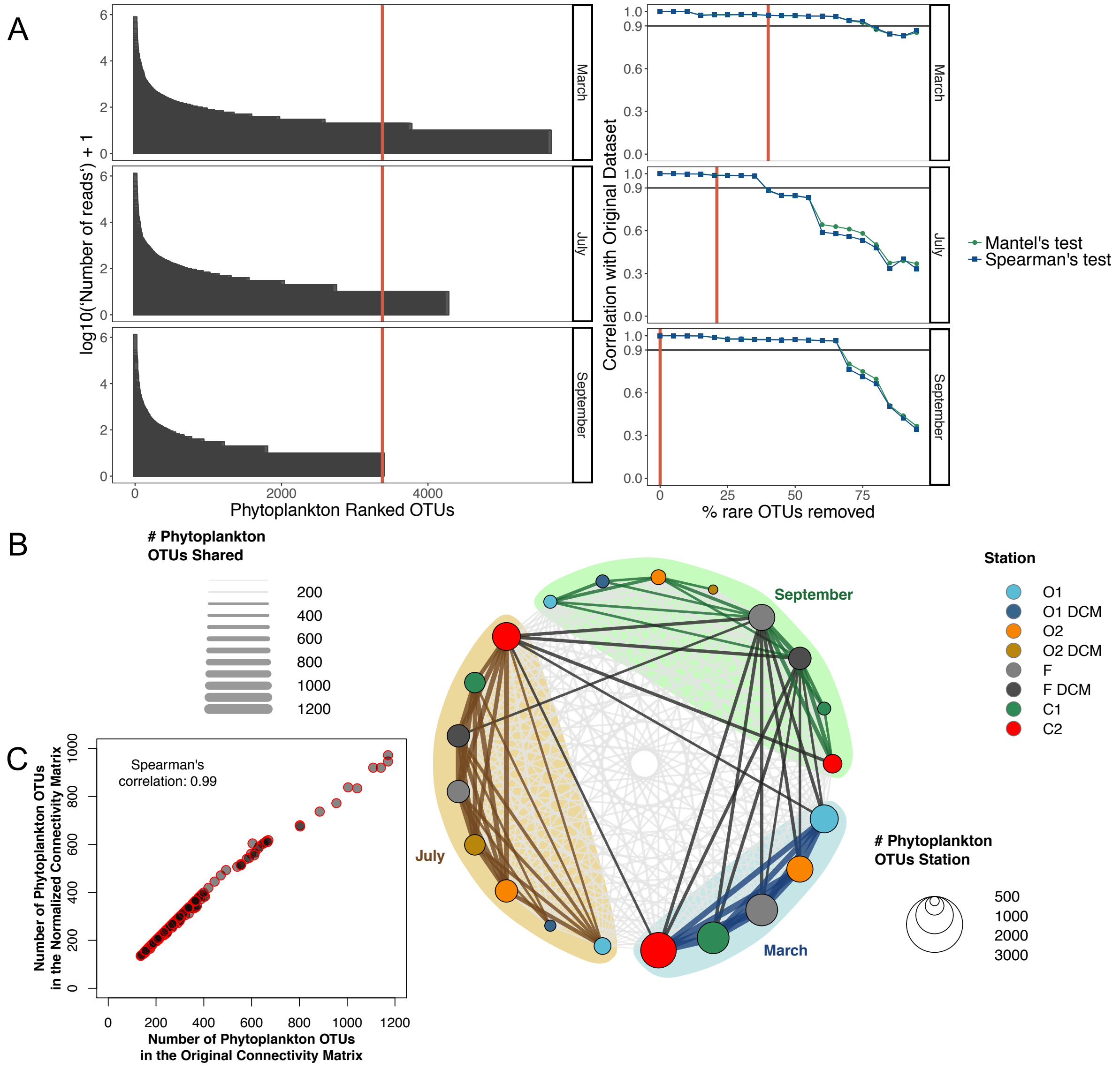

A) At the Left, OTUs of each season were sorted according to their rank abundance, the abundance of OTUs (each bar in the plot) was transformed (log10(x)+1) for the sake of visual representation. We selected a threshold of 3377 OTUs (corresponding to the total number of OTUs retrieved in September, the season with the lowest richness) and kept the first 3377 most abundant OTUs in each season, and discarded the rest. At the right, is represented the effect that

an increasing removal of the less abundant OTUs have on the initial biodiversity patterns of the original dataset. We computed a community similarity matrix of all samples in each season using the Bray-Curtis distance [4]. Using the same methodology, we computed community similarity matrices for sub-datasets with an increasing removal of OTUs. The correlation between the matrices of the original datasets and the sub-datasets was measured based on the spearman correlation and the Mantel test. The threshold selected (3377 final OTUs) represented distinct proportion of OTUs removed in each dataset (March: 40%, July: 21% and September: 0%), however at this threshold the correlation between the original and sub-datasets were high (Mantel and Spearman > 0.9), supposing that the removal of the OTUs under this threshold had low effect on the diversity patterns of eukaryotic phytoplankton.

B) OTU-connectivity of the sub-dataset with a curated number of OTUs by seasons. OTU-connectivity is represented as a network. Nodes size represents the number of OTUs in each station (nodes color) of each season; edge size represents the number of OTUs shared between stations while their colour represents: low connectivity (white, < 300 OTUs), intra- (coloured) or cross- (black) season connectivity. This figure can be compared to the Figure 4 from our paper.

C) OTU-connectivity represent the number of OTU shared between all stations. We compared the OTU-connectivity of the original and curated dataset, i.e. the number of OTUs shared by all pairs of station in the original and curated dataset. The correlation between the two matrices was calculated with the Spearman rank correlation. The good correlation (R^2 = 0.99) indicates that OTU-connectivity patterns in the original dataset and the curated dataset were very similar, despite a distinct total number of OTUs by season.

Figure S4: Statistical tests to detail the OTU-connectivity patterns of eukaryotic phytoplankton in the Iroise Sea in 2015. Graphics represents boxplots of the number of phytoplankton OTUs shared by edge in the network presented in Figure 4. A) Variability of edge weight (# of phytoplankton OTUs shared) among edges occurring across and within seasons (total of 630 edges). B) Variability of edge weight occurring within each season (198 edges within seasons). C) Variability of edges weight occurring within seasons and involving the frontal station *versus* edges between other stations. D) Variability of edges weight occurring across seasons and involving the frontal station *versus* edges between other stations. The number of edges in each category is represented under the boxplots. *** represents a statistical difference with a p.value < 0.0001 according to the Kruskal-Wallis test.

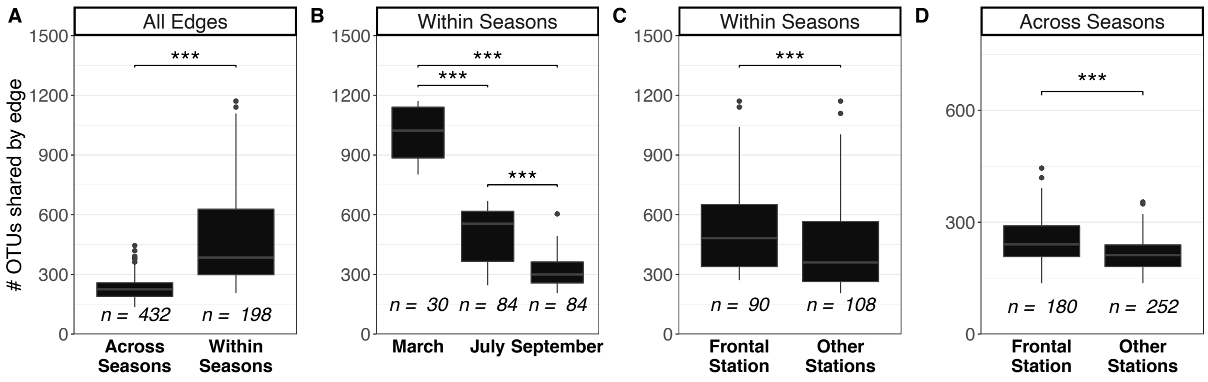

A) The number of OTUs shared by stations within seasons is significantly higher than across seasons.

B) The number of OTUs shared by stations within seasons is significantly decreasing from March to September.

C) Within seasons, the number of OTUs shared by the frontal station is significantly higher than in other stations.

D) Across seasons, the number of OTUs shared by the frontal station is significantly higher than in other stations.

Figure S5: Null-model approach to infer the dominant ecological processes driving the community composition of eukaryotic phytoplankton of the Iroise Sea in 2015. The first figure is modified from Zhou and Ning (2017). We secondly give more details about the construction of Figure 4B of the original paper. Finally, we detail the link between the phylogenetic distance and trait distance of eukaryotic phytoplankton.

We followed the approach of Stegen et al. (2013), further reviewed by Zhou and Ning (2017). We used non-weighted metrics because our metabarcoding of eukaryotic phytoplankton is semi-quantitative. Two custom R scripts were used. The script from Stegen et al. (2013) computes an index based on the observed phylogenetic turnover and 999 null-models of phylogenetic turnover, i.e. the Nearest Taxon Index ßNTI. The ß-Mean-Nearest Taxon Distance (ßMNTD) is first computed using the *comdistnt()* function of R package "Picante" [7], with the parameter *abundance.weighted = FALSE*. The *comdistnt()* function computes a metric of community similarity weighted by the phylogenetic distance between eukaryotic OTUs. The information about OTUs phylogeny was retrieved following the methods detailed in [8] (see below for more information). The 999 null-models are computed with the same method but with an additional random shuffling of the names of the OTUs in the phylogenetic distance provided to the *comdistnt()* function. Then, the ßNTI is the "difference between the observed ßMNTD and the mean of the null distribution, it is measured in units of standard deviation (of the null distribution)" [6]. The script from Chase et al. (2011) computes an index based on the observed taxonomic turnover and by 999 null-models of taxonomic turnover, i.e. the Raup-Crick RC metric based on Jaccard's distance (a non-weighted metric). The 999 null-models are also computed with Jaccard's distance but this time it is the presence absence of all OTUs across each pairwise community comparison that is randomized. The RC metric is then computed by counting how many values within the null-models are bigger than the observed values for all pairwise community comparisons, dividing these counts by the number of null-models, and normalizing the value between -1 and 1 [9]. The approach and how to interpret the ßNTI and RC metrics are summarized in the following figure. The definitions of the ecological processes resulting from the interpretation of this approach are the one given in Zhou and Ning (2017).

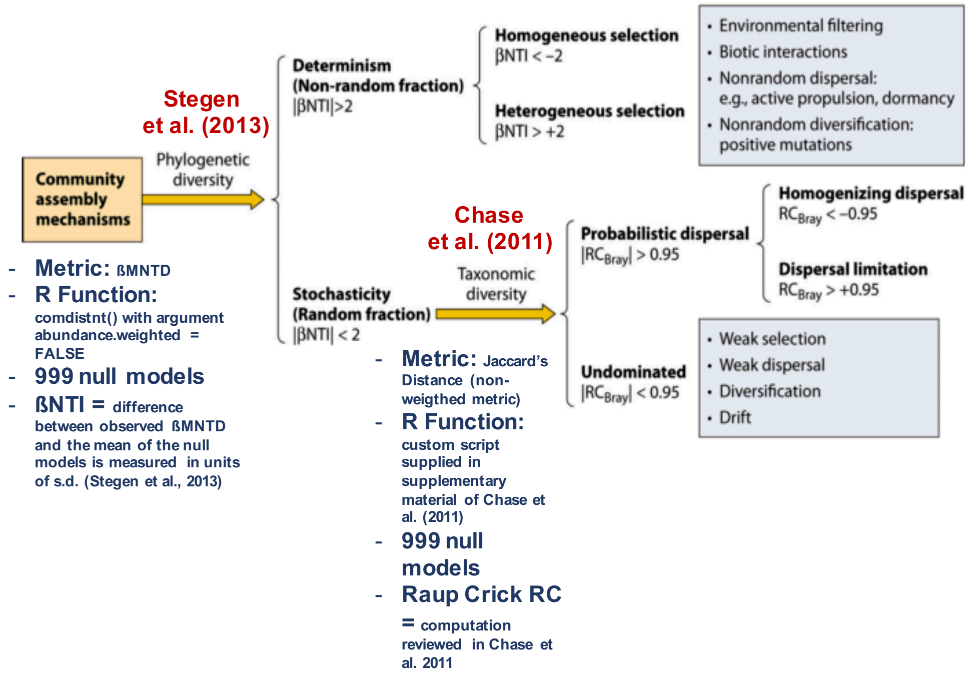

To assess the dominant ecological processes between each pair of communities, two matrices are computed (one for ßNTI one for RC). Each matrix is constituted of 184 * 184 = 33 856 pairwise community comparisons (PCC), each matrix is symmetrical ([C1;C2] = [C2;C1]) so we discarded the upper triangle of each matrix, resulting in 17020 PCC. We converted the matrices into lists (3 columns: [id of community 1], [id of community 2], [ßNTI or RC]) and merged them into a single list (4 columns: [id of community 1], [id of community 2], [ßNTI] and [RC]). We discarded the PCC of the same community (=> 16 836 PCC) but also PCC of different size-fractions because their difference is explained by a methodological separation not by an ecological process (=> 5551 PCC). Every PCC was characterized by an ecological process using the values of ßNTI and RC and following the interpretation illustrated above. Out of 5551 PCC, 5545 were explained by Homogenous Selection, 3 by Dispersal Limitation, 2 by Homogenizing Dispersal and 1 showing an Undominated Scenario (interpreted as ecological drift). As explained in the paper, all PCC explained by anything but Homogenous Selection were found in PCC of micro-phytoplankton. For a PCC to be dominated by anything but selection supposes that the |ßNTI| is < 2 and the observed phylogenetic turnover was significantly different than expected by chance [6]. The similarity or dissimilarity within this PCC is thus explained by dispersal or stochasticity more than by selection [5]. The dominant ecological processes between phytoplankton communities were represented in a network with the same structure as the network for OTU-connectivity

(see Figure 4 replicated here below), with edge colors representing the dominant ecological processes explaining between two communities of eukaryotic phytoplankton (nodes). The structure of this network allows to characterize 21 * 21 = 441 PCC. The original 5551 PCC were summarized in this network, we put emphasis on the 6 PCC that were not dominated homogenous selection, despite the fact that sometimes this process did not explain the PCC across all the size-fractions and replicates of these communities. The size of the edge linking ‘C1 in July’ and ‘F in September’ was doubled because an ecological process other the Homogenous Selection appeared more than once (across replicates of the micro-plankton).

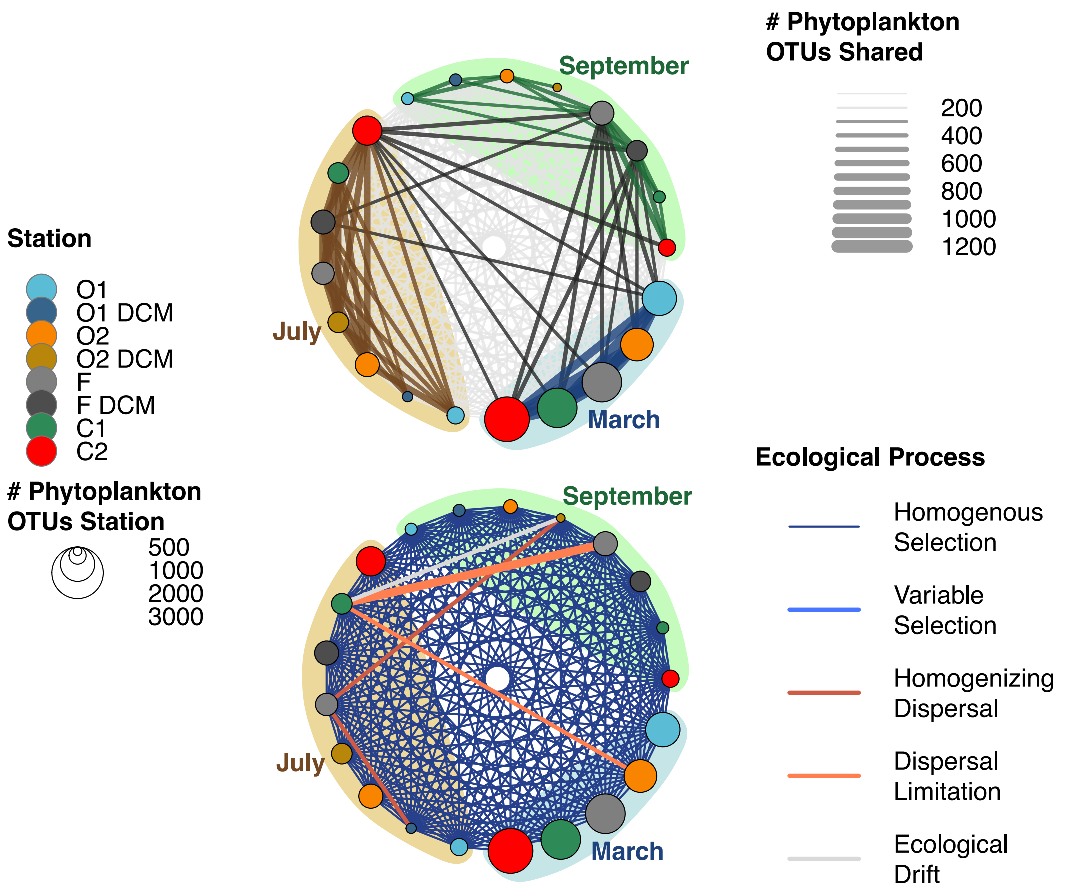

A strong hypothesis behind this null-model approach is that phylogeny is used as a proxy for niche divergence between taxa, phylogenetic turnover is then used to infer niche-based Selection. Before applying the approach, we tested this hypothesis for eukaryotic phytoplankton OTUs. In a previous work we annotated traits to protistan OTUs that represent their ecological strategy or niche [10]. In the dataset of the Iroise Sea, we recovered 7106 eukaryotic phytoplankton OTUs (out of 10 597) well annotated with the 13 following traits: SizeMin, SizeMax, Cell Cover, Cell Shape, Presence of Spicule, Cell

Symmetry, Cell Polarity, Coloniality, Motility, Chloroplast Origin, Ingestion Method, Symbiosis Type and Resting Stage during the life cycle. Based on the trait table (7106 OTUs x 13 traits) we computed a pairwise similarity of OTUs based on their traits using the Gower distance [4], resulting in a matrix of 7106 * 7106 = 50 495 236 pairwise OTU comparisons. A similar pairwise matrix of phylogenetic distance was computed with R following Callahan et al., (2016), the method comprises a sequence multiple-alignment (here eukaryotic phytoplankton V4 sequences of the 18S rDNA), phylogenetic distance computation with the R function *dist.ml()* of R package "Phangorn" [11], and the computation of a neighbor-joining tree fitted to a GTR+G+I maximum likelihood tree. Because it was computationally impossible to study the correlation between phylogenetic and trait distance across all 50 495 236 pairwise OTUs comparisons, we sampled 100 OTUs from the initial 7106 and studied the correlation between trait and phylogenetic similarity across the 100 x 100 = 10.000 pairwise OTUs comparisons, we repeated this process 999 times to have a good approximation of the correlation between the two variables. The following figure represents the pairwise comparisons for one draw of 100 OTUs, superimposed on the graph are the average linear model from all 999 draws (in red, the average linear model and coefficients) and the average correlation estimated with the Pearson correlation test. The average correlation between OTUs trait and phylogenetic similarity was high (R^2 = 0.39) highlighting that phylogenetic distance was a good indicator of the ecological niche of eukaryotic phytoplankton OTUs. Phylogenetic turnover was thus a good proxy for niche-based selection. Trait approaches might be better at approximating niche-based selection, however we did not combine the method of Stegen et al. (2013) with our trait approach because not all OTUs were annotated with traits while all carried phylogenetic information. Combining both null-models and trait approaches represent an interesting perspective for quantifying community assembly processes, nevertheless this goal was beyond the scope of our study.

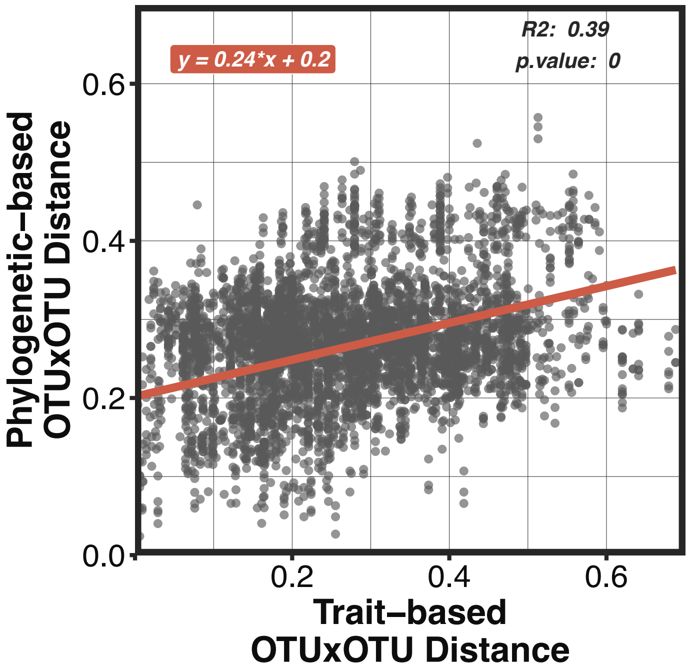

y = 0.24*x + 0.2
R2: 0.39
p.value: 0
0.6
0.4
0.2
0.0
0.2
0.4
0.6
Phylogenetic−based
OTUxOTU Distance
Trait−based
OTUxOTU Distance

Figure S6: Synthesis of eukaryotic phytoplankton ecological strategies based on 7106 OTUs annotated with 13 functional traits and sampled in the Iroise Sea in 2015. The 13 traits were: SizeMin, SizeMax, Cell Cover, Cell Shape, Presence of Spicule, Cell Symmetry, Cell Polarity, Coloniality, Motility, Chloroplast Origin, Ingestion Method, Symbiosis Type and Resting Stage during the life cycle. A) Based on the trait table (7106 OTUs x 13 traits) we first constructed a Hierarchical clustering tree of eukaryotic phytoplankton OTUs using the Gower distance and the complete linkage algorithm [4]. The dendrogram representation showed 9 clusters that were considered as ecological strategies. B) To characterize the ecological strategies, we investigated the trait and modalities prevalent among the OTUs contained in each cluster/ecological strategy. C) For the same reason, we studied the proportion of OTUs' taxonomic ranks inside each cluster/ecological strategy. We evidenced the following ecological strategies (characterized by their trait or when possible with the dominant taxonomy):

- Ecological Strategy 1: Rhizaria
- Ecological Strategy 2: Elongated Diatoms
- Ecological Strategy 3: Colonial Diatoms with Spicules
- Ecological Strategy 4: Round Colonial Diatoms
- Ecological Strategy 5: Colonial Micro-flageallates
- Ecological Strategy 6: Elongated, Mixotrophic Dinoflagellates
- Ecological Strategy 7: Calcareous Micro-flagellates
- Ecological Strategy 8: Micro-flagellates with Spicules
- Ecological Strategy 9: Small-flagellates

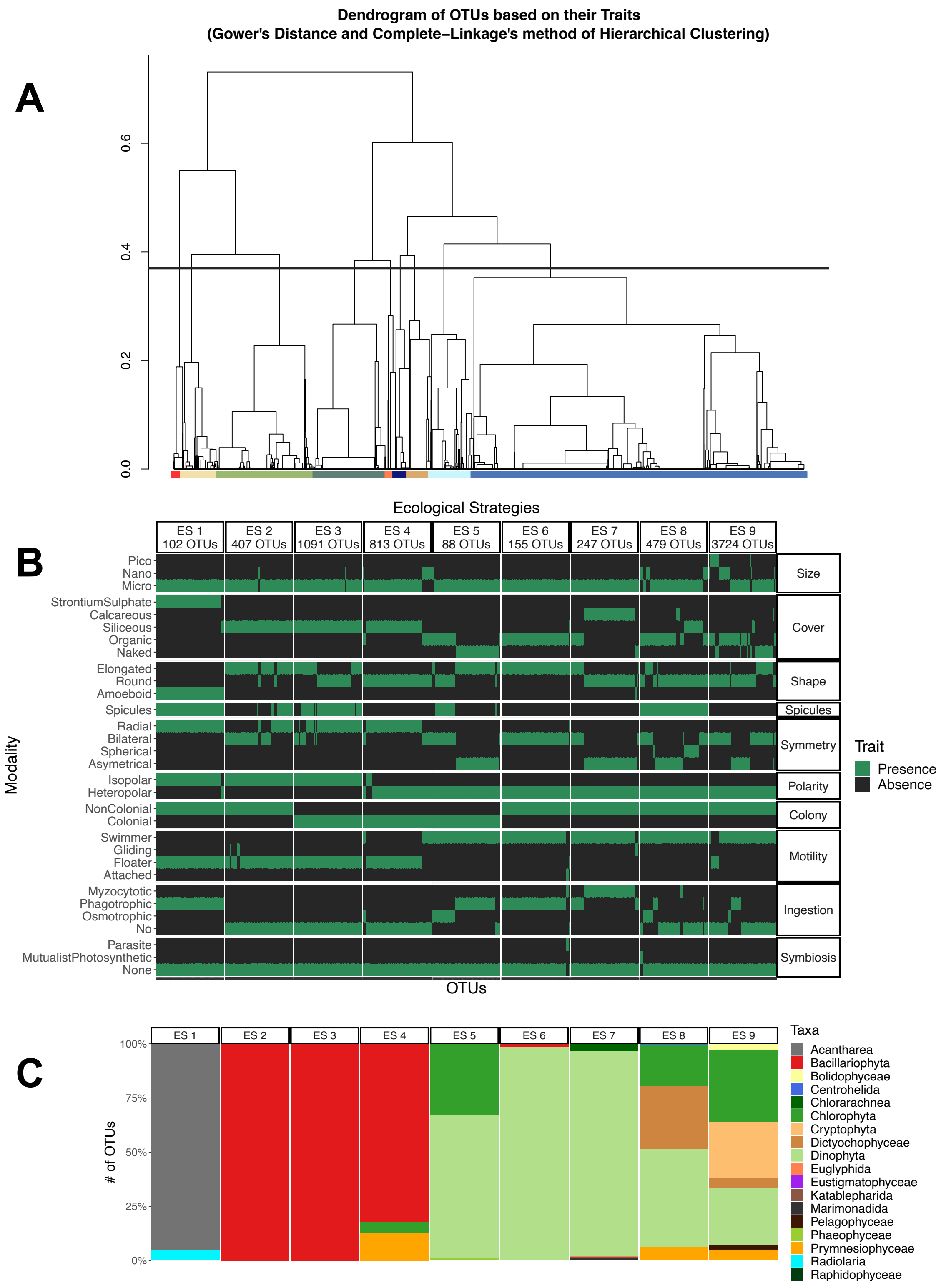

Dendrogram of OTUs based on their Traits
(Gower's Distance and Complete–Linkage's method of Hierarchical Clustering)
A
0.0
0.2
0.4
0.6
B
Ecological Strategies
ES 1 102 OTUs
ES 2 407 OTUs
ES 3 1091 OTUs
ES 4 813 OTUs
ES 5 88 OTUs
ES 6 155 OTUs
ES 7 247 OTUs
ES 8 479 OTUs
ES 9 3724 OTUs
Modality
Pico
Nano
Micro
StrontiumSulphate
Calcareous
Siliceous
Organic
Naked
Elongated
Round
Amoeboid
Spicules
Radial
Bilateral
Spherical
Asymetrical
Isopolar
Heteropolar
NonColonial
Colonial
Swimmer
Gliding
Floater
Attached
Myzocytotic
Phagotrophic
Osmotrophic
No
Parasite
MutualistPhotosynthetic
None
Size
Cover
Shape
Spicules
Symmetry
Polarity
Colony
Motility
Ingestion
Symbiosis
OTUs
Trait
Presence
Absence
C
ES 1
ES 2
ES 3
ES 4
ES 5
ES 6
ES 7
ES 8
ES 9
# of OTUs
0%
25%
50%
75%
100%
Taxa
Acantharea
Bacillariophyta
Bolidophyceae
Centrohelida
Chlorarachnea
Chlorophyta
Cryptophyta
Dictyochophyceae
Dinophyta
Euglyphida
Eustigmatophyceae
Katablepharida
Marimonadida
Pelagophyceae
Phaeophyceae
Prymnesiophyceae
Radiolaria
Raphidophyceae

References

1. Oksanen, J. *et al.* vegan: Community Ecology Package. (2018).
2. Hsieh, T. C., Ma, K. H. & Chao, A. iNEXT: an R package for rarefaction and extrapolation of species diversity (Hill numbers). *Methods Ecol. Evol.* **7,** 1451–1456 (2016).
3. Gobet, A., Quince, C. & Ramette, A. Multivariate cutoff level analysis (MultiCoLA) of large community data sets. *Nucleic Acids Res.* **38,** (2010).
4. Legendre, P. & Legendre, L. *Numerical Ecology. Third English Edition*. (Elsevier, 2012).
5. Zhou, J. & Ning, D. Stochastic Community Assembly : Does It Matter in Microbial Ecology? *Microbiol. Mol. Biol. Rev.* **81,** 1–32 (2017).
6. Stegen, J. C. *et al.* Quantifying community assembly processes and identifying features that impose them. *ISME J.* **7,** 2069–2079 (2013).
7. Kembel, S. W. *et al.* Picante: R tools for integrating phylogenies and ecology. *Bioinformatics* **26,** 1463–1464 (2010).
8. Callahan, B. J., Sankaran, K., Fukuyama, J. A., McMurdie, P. J. & Holmes, S. P. Bioconductor workflow for microbiome data analysis: From raw reads to community analyses [version 1; referees: 3 approved]. *F1000Research* **5,** 1–49 (2016).
9. Chase, J. M., Kraft, N. J. B., Smith, K. G., Vellend, M. & Inouye, B. D. Using null models to disentangle variation in community dissimilarity from variation in α-diversity. *Ecosphere* **2,** (2011).
10. Ramond, P. *et al.* Coupling between taxonomic and functional diversity in protistan coastal communities. *Environ. Microbiol.* **21,** 730–749 (2019).
11. Schliep, K. P. phangorn: Phylogenetic analysis in R. *Bioinformatics* **27,** 592–593 (2011).
